# Detection of kinematic abnormalities in persons with knee osteoarthritis using markerless motion capture during functional movement screen and daily activities

**DOI:** 10.3389/fbioe.2024.1325339

**Published:** 2024-02-05

**Authors:** Fei Wang, Rui Jia, Xiuming He, Jing Wang, Peng Zeng, Hong Hong, Jiang Jiang, Hongtao Zhang, Jianyi Li

**Affiliations:** ^1^ Department of Anatomy, Guangdong Provincial Key Laboratory of Digital Medicine and Biomechanics, Guangdong Engineering Research Center for Translation of Medical 3D Printing Application, School of Basic Medical Sciences, Southern Medical University, Guangzhou, China; ^2^ Nanchang Medical College, Nanchang, China; ^3^ Department of Rehabilitation Medicine, Guangdong Provincial People’s Hospital (Guangdong Academy of Medical Sciences), Southern Medical University, Guangzhou, China; ^4^ Zhongshan Torch Development Zone People’s Hospital, Zhongshan, China

**Keywords:** knee osteoarthritis, kinematic abnormalities, functional movement screen, markerless motion capture, objective evaluation

## Abstract

**Background:** The functional movement screen (FMS) has been used to identify deficiencies in neuromuscular capabilities and balance among athletes. However, its effectiveness in detecting movement anomalies within the population afflicted by knee osteoarthritis (KOA), particularly through the application of a family-oriented objective assessment technique, remains unexplored. The objective of this study is to investigate the sensitivity of the FMS and daily activities in identifying kinematic abnormalities in KOA people employing a markerless motion capture system.

**Methods:** A total of 45 persons, presenting various Kellgren–Lawrence grades of KOA, along with 15 healthy controls, completed five tasks of the FMS (deep squat, hurdle step, and in-line lunge) and daily activities (walking and sit-to-stand), which were recorded using the markerless motion capture system. The kinematic waveforms and discrete parameters were subjected to comparative analysis.

**Results:** Notably, the FMS exhibited greater sensitivity compared to daily activities, with knee flexion, trunk sagittal, and trunk frontal angles during in-line lunge emerging as the most responsive indicators.

**Conclusion:** The knee flexion, trunk sagittal, and trunk frontal angles during in-line lunge assessed via the markerless motion capture technique hold promise as potential indicators for the objective assessment of KOA.

## 1 Introduction

Objective evaluations play a pivotal role in assessing the therapeutic effectiveness of knee osteoarthritis (KOA) treatments and in monitoring disease progression for precise adjustments in treatment strategies (Bellamy et al., 2009). However, the existing methods of KOA assessment exhibit certain limitations. Radiography lacks the required sensitivity to discern short-term knee alterations (Hayashi et al., 2016). Magnetic resonance imaging is expensive and thus impractical for daily monitoring of persons with KOA (PwKOA) (van Helvoort et al., 2021). Patient-reported outcome measures, when utilized outside clinical settings, tend to be highly subjective and can exhibit a ceiling effect (van der Straaten et al., 2020). Therefore, a need persists for a simple and dependable measurement technique to evaluate KOA objectively.

Recent studies have highlighted the decline in motor and balance capabilities experienced by PwKOA, thereby amplifying the research emphasis on quantitatively assessing exercise and balance functions associated with KOA (Lee et al., 2019; Zeng et al., 2022). Gait analysis has emerged as the most widely used approach for this purpose (Mills et al., 2013). Additionally, some researchers have explored the potential utility of the timed up-and-go test, which involves multiple tasks representative of daily activities, for KOA evaluation (Hsieh et al., 2020). However, PwKOA are more prone to exhibiting compensatory movement strategies when confronted with challenging functional tasks (van der Straaten et al., 2018).

The functional movement screen (FMS) is a systematic screening tool capable of identifying asymmetries or compensatory strategies within the movement patterns of an individual (Smith and Hanlon, 2017; Lee et al., 2019). Although the FMS has been successfully employed to detect deficits in neuromuscular capacity and balance among athletes (Moran et al., 2017; Lee et al., 2018; Suzuki et al., 2022), its potential in identifying movement anomalies within PwKOA remains unexplored. Furthermore, it remains uncertain whether the FMS exhibits greater sensitivity compared to routine daily activities.

Quantitative motion evaluations of KOA have traditionally relied on marker-based motion capture systems, which are not only expensive and time-consuming, but also demand specialized expertise (Topley and Richards, 2020; Vitali and Perkins, 2020). Given that conservative KOA treatment is often administered within the community or at home, a timely and family-oriented objective monitoring technique is required. Owing to its cost-effectiveness and unobtrusive motion assessment capabilities (Takeda et al., 2021), markerless motion capture technology has emerged as a promising alternative. Nonetheless, its potential to detect abnormal motor performance in PwKOA remains largely unexplored.

Therefore, the primary objective of this study is to determine the kinematic parameters that most accurately distinguish between persons with varying Kellgren–Lawrence (K–L) grades of KOA and healthy controls (HC) during FMS (deep squat, hurdle step, and in-line lunge) and daily activities (walking and sit-to-stand) when employing a markerless motion capture system.

## 2 Methods

### 2.1 Participants

Fifteen HC from local communities and 45 PwKOA from a local hospital were recruited for the study. The PwKOA were divided into three groups: mild (K–L grade 1), moderate (K–L grade 2), and severe (K–L grade 3/4). Each group contained 15 people. For the purposes of this study, K–L grades 3 and 4 were considered as a single group (Fukaya et al., 2019; Ismailidis et al., 2020). The selected patients had confirmed imaging findings indicative of primary KOA. The HC group exhibited a balanced gender distribution and ages ranging between 45 and 75 years. Exclusion criteria included the existence of a history of knee surgery, unresolved lower extremity joint injury, body mass index >30 kg/m^2^, walker use, and inability to adhere to the test protocol.

Ethical approval for this study was obtained from the ethics committee of Zhongshan Torch Development Zone People’s Hospital (2022-0001). All participants provided written informed consent.

### 2.2 Data collection

First, all participants completed the Western Ontario and McMaster University Osteoarthritis Index (WOMAC) questionnaire and then completed five tasks of the FMS (deep squat, hurdle step, and in-line lunge) and daily activities (walking and sit-to-stand). Before the formal test, the participants were trained to ensure that their actions were conducted in accordance with the standard. The instructions provided to the participants are listed in Table 1. All tasks were recorded using a markerless motion capture system. Each participant performed three times for each task, and the average of the three tests was analyzed. The parameters of interest were obtained from the dominant and affected sides of the participants. In cases of bilateral PwKOA, the affected side was defined as the more painful one; in cases of equal pain, the dominant side was chosen. The parameters of interest encompassed the trunk frontal angle, trunk, hip, and knee sagittal angles, and medial-lateral displacement of the center of mass (COM ML displacement). Balance control proficiency was primarily indicated by the trunk frontal angle, trunk sagittal angle, and COM ML displacement, whereas movement performance was mainly reflected by the hip and knee sagittal angles. Table 2 presents a comprehensive list of the parameters of interest.

**TABLE 1:** Instructions given to the participants.

Task	Instructions to participant
Deep squat	1) Stand with your feet shoulder-width apart and your toes facing forward in line with the mark. Hold the pole above your head with both hands. 2) Keep your trunk upright and your feet and pole in position, squat as deep as you can, then move at your preferred speed back to the starting position
Hurdle step	1) Stand with your feet together and your toes facing forward in line with the mark. Hold the pole behind your neck with both hands. 2) With your trunk upright, bend your dominant (or affected) lower limb over the rail so that the heel touches the floor (without force) and return to the starting position, maintaining alignment between the foot, knee, and hip as much as possible
In-line lunge	1) Position your dominant (affected) heel at the intended mark line, align the other toe with the initial mark line, and extend both knees. 2) Keep your trunk upright and stretch your arms horizontally. 3) Lower your back knee to the heel of your front foot, and then return to the standing position
Sit-to-stand	1) Sit in the chair and lean back with your hands on the armrests. 2) When you hear the “stand up” command, stand up as you would normally do
Walking	1) Stand with your toes facing forward in line with the mark. 2) When you hear the “go” command, walk at a comfortable speed, as you would normally do, until you have passed the stopping line

**TABLE 2:** Parameters measured for each of the five tasks performed.

	Deep squat	Hurdle step	In-line lunge	Sit-to-stand	Walking
Knee sagittal angel waveform	√	√	√		√
Peak knee flexion angle	√	√	√		√
Hip sagittal angel waveform	√	√	√		√
Peak hip flexion or extension angle	√	√	√		√
Trunk sagittal angle waveform	√	√	√		√
Peak trunk sagittal angle	√	√	√	√	√
Trunk frontal angle waveform	√	√	√		√
Peak trunk frontal angle	√	√	√	√	√
COM ML displacement waveform	√	√	√		
Peak COM ML displacement	√	√	√	√	
Gait phase					√
Stride frequency					√
Step length					√

Image data were acquired using a markerless motion capture system (Fast-Move Ltd., China) equipped with four standard video cameras with a resolution of 1,624 × 1,240, which were positioned around the testing area with an angle of approximately 90° between the main optical axes of every pair of adjacent cameras. The sampling rate was 30 Hz. Fast-Move 3D Motion software (version 1.2, Fast-Move Ltd., China) was employed for data processing. An artificial intelligence-based joint-point recognition function automatically analyzed the video images using a 21-point body model (Hay, 1993). The 21 key points included the head, chin, neck, bilateral shoulder, elbow, wrist, hand, hip, knee, ankle, heel and big toe. The sagittal joint angle of the lower limbs, trunk tilt angle, and three-dimensional coordinates of the center of mass were calculated based on the three-dimensional coordinates of the aforementioned 21 points.

Pictures related to data collection and processing are provided in the Supplementary Material.

### 2.3 Data normalization

The kinematic parameters were normalized in terms of time, ranging from 0% to 100%. In the cases of the deep squat, hurdle step, and in-line lunge, normalization occurred between two consecutive points at which the knee reached full extension, using the rate of change of the knee sagittal angle. The sit-to-stand was normalized from the time the trunk started to lean forward to the time the subject stood up straight using the rate of change of the trunk sagittal angle and the up-and-down displacement of the center of mass. For the walking stride, normalization spanned from one heel strike to the next.

### 2.4 Statistical analysis

Statistical analyses were conducted using PASS (version 15.0, NCSS, United States), MATLAB (version 2021a, MathWorks, Inc., United States), and SPSS (version 22.0, IBM Corporation, United States).

The required sample size was calculated based on the group differences observed in the discrete parameters reported by Nüesch *et al.* (2021). To detect an expected difference in knee angle with an effect size of at least 1.12% and 80% power at a significance level of 5%, a minimum of 14 participants was deemed necessary for each group.

One-dimensional statistical parametric or nonparametric mapping (SPM or SnPM) was used to compare the kinematic waveforms of HC and PwKOA in different groups (Pataky et al., 2013; Papi et al., 2020). Depending on the adherence of the data to normality, either a parametric or nonparametric one-way ANOVA was used to compare the time-varying kinematic parameters. To test the null hypothesis stating that no differences exist between the groups, a critical threshold that only 5% of the smooth random curves would be expected to intersect was calculated. In instances of observed significant differences, *post hoc* analyses encompassing parametric or nonparametric two-tailed two-sample t-tests were conducted on HC and PwKOA across various groups. Statistical significance materialized when the SPM curves (SPM{X2}, SnPM{X2}, SPM{t}, or SnPM{t}) intersected the critical threshold at any given node. In all SnPM tests, the iteration count was set at 10,000.

Discrete kinematic parameters were compared between HC and PwKOA across distinct groups through either one-way ANOVA or Wilcoxon’s nonparametric test, contingent upon the adherence of the data to normality. Subsequent *post hoc* analyses were conducted for identified significant differences. The significance level was set at *p* < 0.05.

## 3 Results

### 3.1 Participants

Table 3 presents the characteristics of the participants, all of whom were right-handed. Notably, the PwKOA were significantly older than the HC group (*p* < 0.01). The substantially lower WOMAC scores among the HC confirmed the absence of knee-related pain or disability.

**TABLE 3:** Characterization of the participants.

	HC (n = 15)	Mild (n = 15)	Moderate (n = 15)	Severe (n = 15)
Male/female	3/12	3/12	3/12	4/11
Age (years)	52.67 ± 4.65	57.00 ± 6.87^a^	60.80 ± 5.43^a^	64.87 ± 5.67^a^ ^,^ ^b^
Height (m)	1.56 ± 0.06	1.51 (1.49–1.62)	1.54 ± 0.07	1.57 ± 0.09
Weight (kg)	54.50 (50.40–60.90)	56.32 ± 11.06	59.93 ± 5.23	61.25 ± 12.95
BMI (kg/m^2^)	23.07 ± 3.19	23.40 ± 3.06	25.35 ± 2.03	24.67 ± 3.58
Unilateral/Bilateral	-	13/2	11/4	8/7
WOMAC Total	0.00 (0.00–2.00)	9.00 (6.00–16.00)^a^	26.53 ± 13.85^a^	40.80 ± 28.99^a^
WOMAC Pain	0.00 (0.00–0.00)	3.00 (2.00–9.00)^a^	6.13 ± 3.48^a^	9.60 ± 6.01^a^
WOMAC Stiffness	0.00 (0.00–0.00)	0.00 (0.00–0.00)	0.00 (0.00–4.00)	2.00 (0.00–4.00)^a^
WOMAC Function	0.00 (0.00–0.00)	4.00 (3.00–12.00)	18.27 ± 10.53^a^	29.00 ± 25.02^a^

Values other than gender are expressed as mean ± SD, except where the data are non-normally distributed, in which case they are presented as median (IQR).

^a^ Significant difference between this and the HC, groups.

^b^ Significant difference between this and the mild groups.

### 3.2 SPM analysis

**FIGURE 1:**
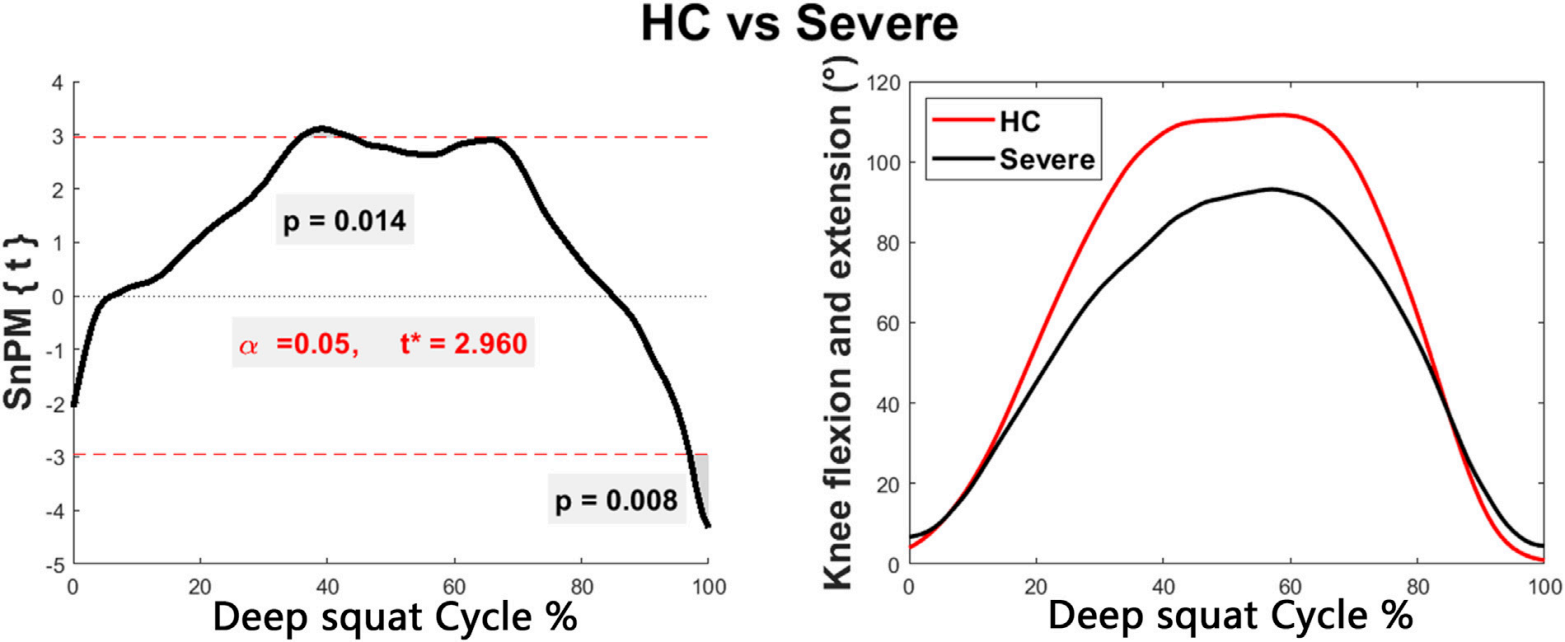
Intergroup disparities of the knee sagittal angle during the deep squat. The curve on the left represents the SnPM{t} curve, and the shaded areas indicate SnPM{t} exceeding the critical threshold (i.e., where statistical differences existed). The curves on the right represent the discriminant angular waveforms (mean) between HC and persons in the severe group.

**FIGURE 2:**
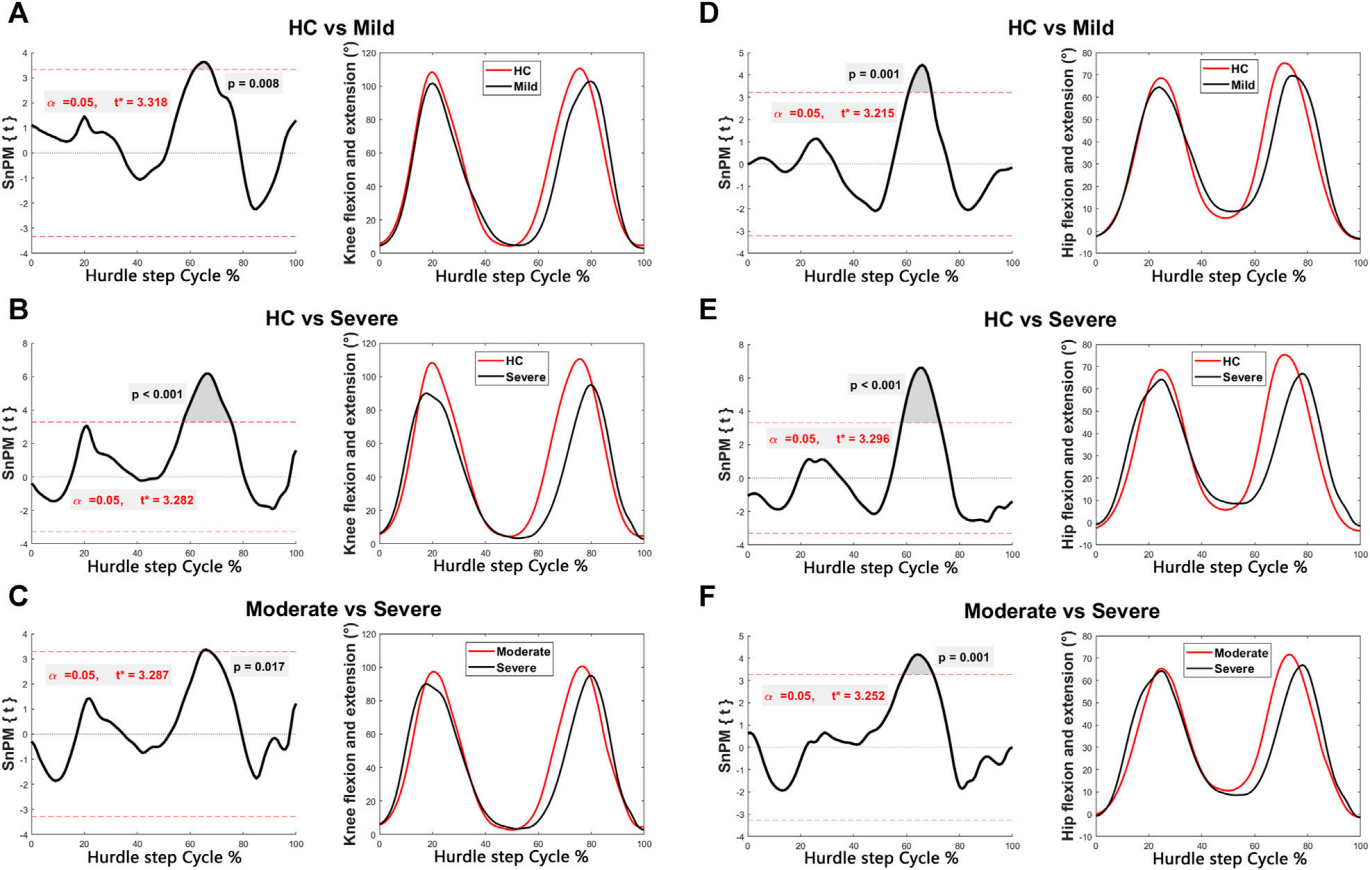
Intergroup disparities of the knee and hip sagittal angles during the hurdle step. The SnPM{t} curves are displayed on the left of the subfigures, and the shaded areas indicate SnPM{t} exceeding the critical threshold (i.e., where statistical differences existed). The discriminant angular waveforms (mean) during the hurdle step are displayed on the right of the subfigures. Difference in knee flexion and extension between **(A)** HC and persons in the mild group; **(B)** HC and persons in the severe group; and **(C)** persons in the moderate and severe groups. Differences in hip flexion and extension between **(D)** HC and persons in the mild group; **(E)** HC and persons in the severe group; and **(F)** persons in the moderate and severe groups.

**FIGURE 3:**
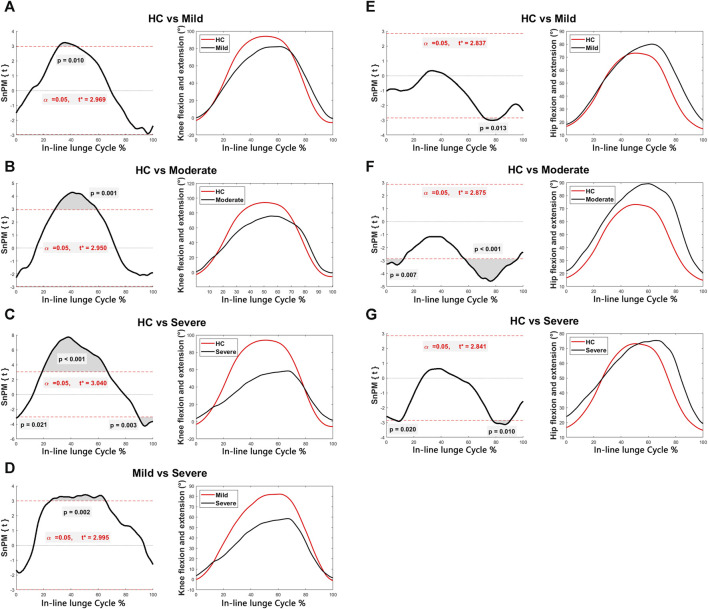
Intergroup disparities of the knee and hip sagittal angles during the in-line lunge. The SnPM{t} curves are displayed on the left of the subfigures, and the shaded areas indicate SnPM{t} exceeding the critical threshold (i.e., where statistical differences existed). The discriminant angular waveforms (mean) during the in-line lunge are displayed on the right of the subfigures. Difference in knee flexion and extension between **(A)** HC and persons in the mild group; **(B)** HC and persons in the moderate group; **(C)** HC and persons in the severe group; and **(D)** persons in the mild and severe groups. Difference in hip flexion and extension between **(E)** HC and persons in the mild group; **(F)** HC and persons in the moderate group; and **(G)** HC and persons in the severe group.

**FIGURE 4:**
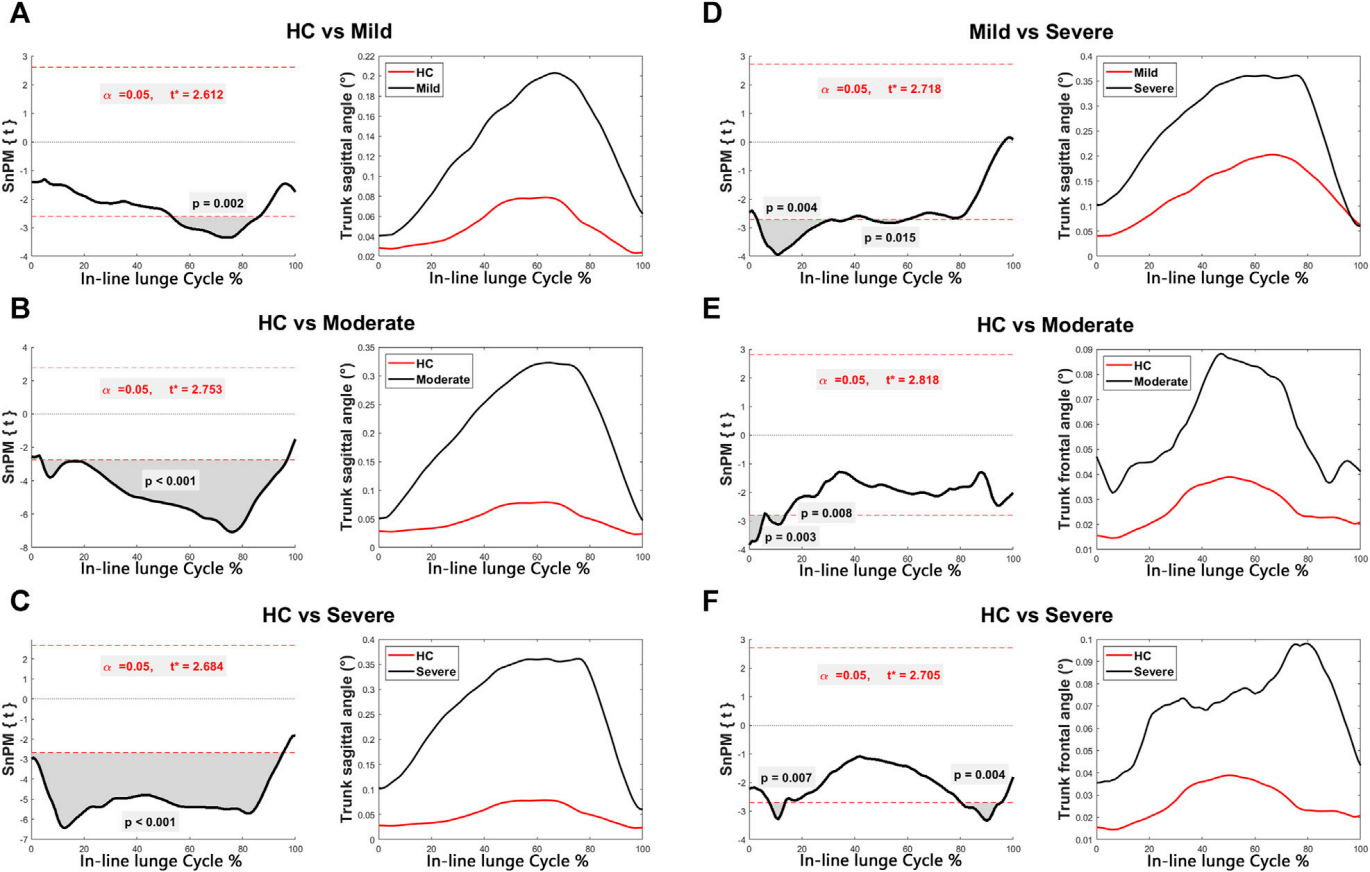
Intergroup disparities of the trunk sagittal and frontal angles during the in-line lunge. The SnPM{t} curves are displayed on the left of the subfigures, and the shaded areas indicate SnPM{t} exceeding the critical threshold (i.e., where statistical differences existed). The discriminant angular waveforms (mean) during the in-line lunge are displayed on the right of the subfigures. Difference in trunk sagittal angle between **(A)** HC and persons in the mild group; **(B)** HC and persons in the moderate group; **(C)** HC and persons in the severe group; and **(D)** persons in the mild and severe groups. Difference in trunk frontal angle between **(E)** HC and persons in the moderate group; and **(F)** HC and persons in the severe group.

Discernible disparities were observed in specific angular waveforms during the FMS, yet no such disparities emerged during daily activities. Results are shown in Figure 1, Figure 2, Figure 3, Figure 4. The SnPM{t} curves are displayed on the left of the subfigures, and the shaded areas indicate SnPM{t} exceeding the critical threshold (i.e., where statistical differences existed). The discriminant angular waveforms (mean) are displayed on the right of the subfigures. Detailed results are described below.

Regarding the deep squat:

•   Participants within the severe group exhibited reduced knee flexion (36%–44%, *P* = 0.014) and increased knee flexion (97%–100%, *P* = 0.008) compared to the HC (Figure 1).

Regarding the hurdle step:

•   Participants within the mild (61%–68%, *P* = 0.008) and severe (57%–76%, *P* < 0.001) groups exhibited reduced knee flexion compared to that of HC (Figures 2A,B).
•   Participants within the severe group exhibited less knee flexion compared to those in the moderate group (65%–68%, *P* = 0.017) (Figure 2C).
•   Participants within the mild (61%–69%, *P* = 0.001) and severe (58%–73%, *P* < 0.001) groups exhibited less hip flexion than that of the HC (Figures 2D,E).
•   Participants within the severe group exhibited less hip flexion compared to those in the moderate group (59%–70%, *P* = 0.001) (Figure 2F).

Regarding the in-line lunge:

•   Participants within the mild (31%–44%, *P* = 0.010), moderate (28%–59%, *P* = 0.001), and severe (19%–67%, *P* < 0.001) groups exhibited reduced knee flexion relative to that of the HC (Figures 3A–C).
•   Subjects within the severe group displayed increased knee flexion compared to that of the HC (0%–1%, *P* = 0.021; 89%–100%, *P* = 0.003) (Figure 3C).
•   Participants within the severe group demonstrated decreased knee flexion compared to those in the mild group (25%–65%, *P* = 0.002) (Figure 3D).
•   The mild (72%–83%, *P* = 0.013), moderate (0%–13%, *P* = 0.007; 57%–96%, *P* < 0.001), and severe (6%–11%, *P* = 0.020; 78%–91%, *P* = 0.010) groups exhibited enhanced hip flexion relative to that of the HC (Figures 3E–G).
•   Participants within the mild (53%–86%, *P* = 0.002), moderate (4%–97%, *P* < 0.001), and severe (0%–95%, *P* < 0.001) groups manifested heightened trunk sagittal angles relative to HC (Figures 4A–C).
•   Participants within the severe group displayed increased trunk sagittal angles compared to those in the mild group (3%–37%, *P* = 0.004; 46%–60%, *P* = 0.015) (Figure 4D).
•   Participants within the moderate (0%–6%, *P* = 0.003; 7%–14%, *P* = 0.008) and severe (8%–14%, *P* = 0.007; 80%–95%, *P* = 0.004) groups demonstrated elevated trunk frontal angles compared to those of the HC (Figures 4E,F).

### 3.3 Discrete kinematic parameter analysis

Regarding the deep squat:

•   The severe group exhibited a lower peak knee flexion angle compared to that of the HC (*P* = 0.001).

Regarding the hurdle step:

•   No significant differences were observed among the parameters of the four groups (*P* > 0.05).

Regarding the in-line lunge:

•   Participants within the moderate (*P* = 0.023) and severe (*P* < 0.001) groups displayed a diminished peak knee flexion angle compared to that of the HC.
•   The severe group exhibited a lower peak knee flexion angle compared to the mild group (P = 0.018).
•   The mild (*P* = 0.008), moderate (*P* < 0.001), and severe groups (*P* < 0.001) demonstrated an elevated peak trunk sagittal angle relative to that of the HC.
•   Participants in the moderate (*P* = 0.023) and severe groups (*P* = 0.002) exhibited a heightened peak trunk sagittal angle compared to those in the mild group.
•   Participants within the moderate (*P* = 0.011) and severe groups (*P* < 0.001) displayed an increased peak trunk frontal angle relative to that of the HC.
•   The severe group exhibited a greater peak trunk frontal angle compared to the mild group (*P* = 0.028).
•   Subjects within the severe group showcased a higher peak COM ML displacement than that of the HC (*P* = 0.010).

Regarding walking:

•   Participants within the moderate (*P* = 0.037) and severe (*P* = 0.009) groups exhibited reduced peak hip extension angles in comparison to those of the HC.
•   Subjects in the severe group demonstrated shorter step lengths than those of the HC (*P* = 0.023).

Regarding sit-to-stand:

•   Participants within the severe group exhibited a higher peak trunk frontal angle than participants in the mild group (P = 0.014) and HC (P < 0.001).

A comprehensive presentation of these outcomes is available in Table 4.

**TABLE 4:** Descriptive statistics for the discrete kinematic parameters in four groups.

Parameter	HC	Mild	Moderate	Severe	P
Deep squat					
Peak knee flexion angle (°)	119.16 ± 7.11	110.17 ± 12.84	110.87 (103.96–121.15)	98.54 (83.56–109.41)^a^	0.004^b^
Peak hip flexion angle (°)	111.04 ± 13.64	112.70 ± 13.47	113.84 ± 9.61	111.30 ± 16.60	0.941
Peak trunk sagittal angle (°)	28.33 ± 10.80	34.04 ± 8.96	34.24 ± 9.84	38.90 ± 11.52	0.059
Peak trunk frontal angle (°)	2.89 ± 1.43	3.08 ± 1.20	4.03 ± 1.85	3.88 ± 1.83	0.138
Peak COM ML displacement (mm)	18.74 ± 8.78	18.96 ± 8.14	20.27 ± 5.81	23.16 ± 11.61	0.500
Hurdle step					
Peak knee flexion angle (°)	119.61 ± 9.73	116.91 ± 14.89	115.70 ± 8.80	107.65 ± 14.07	0.055
Peak hip flexion angle (°)	78.10 ± 9.30	76.91 ± 11.00	78.52 ± 7.95	75.50 ± 7.83	0.802
Peak trunk sagittal angle (°)	9.18 ± 2.92	9.50 ± 2.99	8.25 ± 2.48	8.56 ± 1.98	0.549
Peak trunk frontal angle (°)	6.52 ± 2.19	6.95 ± 2.69	8.05 ± 2.97	9.30 ± 4.57	0.096
Peak COM ML displacement (mm)	34.25 ± 16.80	35.04 ± 11.88	32.10 ± 12.89	40.01 ± 20.45	0.600
In-line lunge					
Peak knee flexion angle (°)	95.62 ± 6.80	87.00 ± 14.55	82.53 ± 13.96^a^	64.30 ± 22.28^a^ ^,^ ^c^	0.000^b^
Peak hip flexion angle (°)	74.55 ± 11.05	85.70 ± 10.21	94.73 ± 19.54	81.28 ± 33.33	0.076
Peak trunk sagittal angle (°)	0.09 ± 0.04	0.20 ± 0.10^a^	0.33 ± 0.12^a^ ^,^ ^c^	0.42 ± 0.18^a^ ^,^ ^c^	0.000^b^
Peak trunk frontal angle (°)	0.05 ± 0.03	0.06 (0.03–0.08)	0.10 (0.07–0.14)^a^	0.11 (0.09–0.15)^a^ ^,^ ^c^	0.000^b^
Peak COM ML displacement (mm)	0.22 ± 0.10	0.27 ± 0.14	0.34 (0.18–0.42)	0.39 (0.28–0.48)^a^	0.012^b^
Sit-to-stand					
Peak trunk sagittal angle (°)	25.41 ± 10.40	26.03 ± 8.47	25.17 ± 6.51	24.14 ± 8.24	0.943
Peak trunk frontal angle (°)	3.81 ± 1.30	4.50 ± 1.53	4.80 ± 1.24	5.90 ± 1.88^a^ ^,^ ^c^	0.004^b^
Peak COM ML displacement (mm)	42.08 ± 27.70	47.22 ± 18.20	53.65 ± 25.19	54.85 ± 31.22	0.508
Walking					
Peak knee flexion angle (°)	51.32 ± 6.29	50.25 ± 4.47	51.96 ± 6.38	44.87 ± 8.91	0.025^b^
Peak hip extension angle (°)	13.30 ± 5.97	11.91 ± 3.97	9.88 ± 2.80^a^	8.96 ± 4.25^a^	0.039^b^
Peak trunk sagittal angle (°)	3.94 ± 1.02	3.81 ± 1.45	3.57 ± 1.39	3.98 ± 1.42	0.832
Peak trunk frontal angle (°)	1.97 ± 0.83	1.69 ± 0.57	2.45 ± 0.86	2.36 ± 1.11	0.072
Gait phase (stance phase)	62.93 ± 2.23	61.93 ± 3.71	61.09 ± 2.79	60.16 ± 3.07	0.084
Stride frequency (steps/min)	117.87 ± 6.53	112.34 ± 9.89	111.33 ± 9.92	111.94 ± 10.92	0.214
Step length (m)	0.37 ± 0.04	0.34 (0.34–0.39)	0.36 ± 0.03	0.31 ± 0.07^a^	0.033^b^

Values are expressed as mean ± SD, except where the data are non-normally distributed, where these data are presented as median (IQR).

^a^ Significant difference between this and the HC, groups.

^c^ Significant difference between this and the mild groups.

^b^ *p*-value <0.05.

## 4 Discussion

This study explored the sensitivity of FMS (deep squat, hurdle step, and in-line lunge) and daily activities (walking and sit-to-stand) for identifying kinematic abnormalities in PwKOA through markerless motion capture. Our findings underscore the higher sensitivity of the FMS over daily activities, with the knee flexion, trunk sagittal angle, and trunk frontal angle during the in-line lunge emerging as the most responsive parameters.

Strategic selection of movements is critical in detecting atypical functional performance in PwKOA. In this study, we chose daily activities (walking and sit-to-stand) and FMS (deep squat, hurdle step, and in-line lunge). FMS comprises seven fundamental movement tests, which were screened using human movement patterns during growth and physical activity (Cook et al., 2014a; Cook et al., 2014b). Given that functional anomalies in PwKOA primarily manifest in the lower limbs, FMS actions such as the deep squat, hurdle step, and in-line lunge, which are inherently linked to lower extremity function and holistic balance control, were deemed suitable for analysis. Concurrently, typical routine activities, such as walking and sit-to-stand, were also considered.

KOA motion tests are subjected to an array of data analysis methodologies. Although discrete parameters, encompassing range of motion and specific point values, have been frequently compared (Nüesch et al., 2021), such a limitation to zero-dimensional scalar parameters disregards an entire measurement domain, potentially obscuring disparities occurring at other instances of the task (Pataky et al., 2016). Consequently, the adoption of SPM to compare kinematic waveforms has gained prominence in motion analysis (Malfait et al., 2016; Sole et al., 2017; Goudriaan et al., 2018). Nevertheless, SPM might inadvertently suppress differences in discrete parameters, given that peaks and troughs may occur at disparate temporal points across individuals (Sole et al., 2017). A prior study underscored the complementary nature of these two statistical methodologies in detecting kinematic deviations (Papi et al., 2020). Accordingly, we combined these two methods to probe abnormal motor balance function in PwKOA comprehensively.

In this study, the application of SPM analysis revealed significant intergroup disparities during the FMS, which accentuated as the K-L grade increased, whereas no such disparities surfaced during walking. During the deep squat and in-line lunge, a reduction in knee flexion was evident in PwKOA, which is consistent with prior research (van der Straaten et al., 2020). Furthermore, we found reductions in the knee and hip flexion during the hurdle step in PwKOA, which have not been previously explored. Reduced knee and hip flexion potentially relate to pain and stiffness; however, further exploration is imperative. Additionally, during the in-line lunge, both the trunk sagittal and frontal angles increased with the occurrence and progression of KOA, which may be attributed to heightened trunk oscillation resulting from compromised balance control (Duffell et al., 2014). In contrast to knee flexion observations, PwKOA demonstrated an augmented hip flexion compared to the HC during the in-line lunge, which can be attributed to increased forward trunk inclination. As the markerless system defined the hip sagittal angle in relation to the sagittal plane formed by the trunk axis and the line connecting the hip and knee, greater trunk inclination leads to an increase in the calculated hip flexion. Prior studies (Ismailidis et al., 2020; Nüesch et al., 2021) revealed that PwKOA exhibited less knee flexion and hip extension than HC during walking, a discrepancy not detected by our markerless motion capture system, indicating the need for improved pose estimation algorithms and data labeling (Wade et al., 2022).

Examination of discrete kinematic parameters unveiled significant intergroup differences during both the FMS and daily activities. Consistent with the SPM analysis outcomes, the knee flexion during the deep squat and the trunk sagittal angle, trunk frontal angle, and knee flexion during the in-line lunge exhibited marked variations. Additionally, during the in-line lunge, discrete parameter analysis detected a significant variance in the peak COM ML displacement between the severe PwKOA group and HC, which was not apparent in the SPM analysis. The increase in peak COM ML displacement further underscored the compromised balance of the PwKOA. During walking, PwKOA evidenced diminished step length and peak hip extension angle relative to the HC, a pattern congruent with extant research findings (Mills et al., 2013; Ismailidis et al., 2021). During the sit-to-stand, the peak trunk frontal angle increased as KOA occurred and progressed, which agrees with the findings of earlier investigations (Sonoo et al., 2019). Although previous investigations have highlighted distinctions in the trunk frontal angle and stride frequency during walking and in the trunk sagittal angle during sit-to-stand using marker-based systems (Sonoo et al., 2019; Ismailidis et al., 2021; Waiteman et al., 2022), these differences were not evident in our study, suggesting the potential of markerless motion capture systems for providing improved detection capability.

Several limitations of this study merit consideration. First, during participant recruitment, the age range of the HC was controlled; however, the HC skewed younger than the PwKOA, potentially introducing a source of bias. Furthermore, gender and the affected side could influence the functional performance of PwKOA. Nonetheless, our analysis did not segregate participants based on these variables, primarily due to insufficient statistical power for sample stratification. Third, SPM analysis was not performed for the sit-to-stand measurements. Although we provided standardized instructions, the participants applied various movement strategies during sit-to-stand. Therefore, the difference in the kinematic waveform did not reflect the difference in KOA movement performance.

## 5 Conclusion

By incorporating the markerless motion capture technique, our study underscored the heightened sensitivity of the FMS over daily activities, with the knee flexion, trunk sagittal angle, and trunk frontal angle during the in-line lunge emerging as promising indicators for objective KOA assessment. The potential effectiveness of these indicators for early KOA screening and their evolution as the disease progresses deserve further exploration within comprehensive cohort studies.

## Data Availability

The raw data supporting the conclusions of this article will be made available by the authors, without undue reservation.

## Abbreviations

FMS, functional movement screen; KOA, knee osteoarthritis; PwKOA, persons with KOA; K–L, Kellgren–Lawrence; HC, healthy controls; BMI, body mass index; WOMAC, Western Ontario and McMaster University Osteoarthritis Index; COM ML displacement, medial-lateral displacement of the center of mass; SPM, statistical parametric mapping; SnPM, statistical nonparametric mapping.
