# Supplementary material for: Detection of kinematic abnormalities in persons with knee osteoarthritis using markerless motion capture during functional movement screen and daily activities
Source: Front Bioeng Biotechnol. 2024 Feb 5;12:1325339. doi: 10.3389/fbioe.2024.1325339 (PMC10875007; doi:10.3389/fbioe.2024.1325339)
Supplement: Supplementary file 1 [file DataSheet1.docx]

Supplementary Material

Clinical data collection and processing

# 1. Pictures of the clinical data acquisition


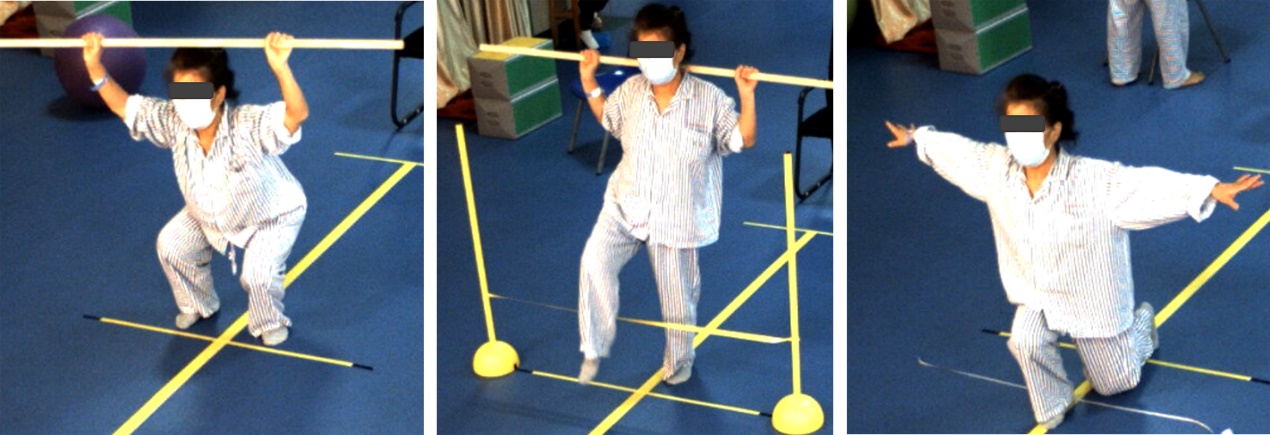


Test of FMS (including deep squat, hurdle step, and in-line lunge)


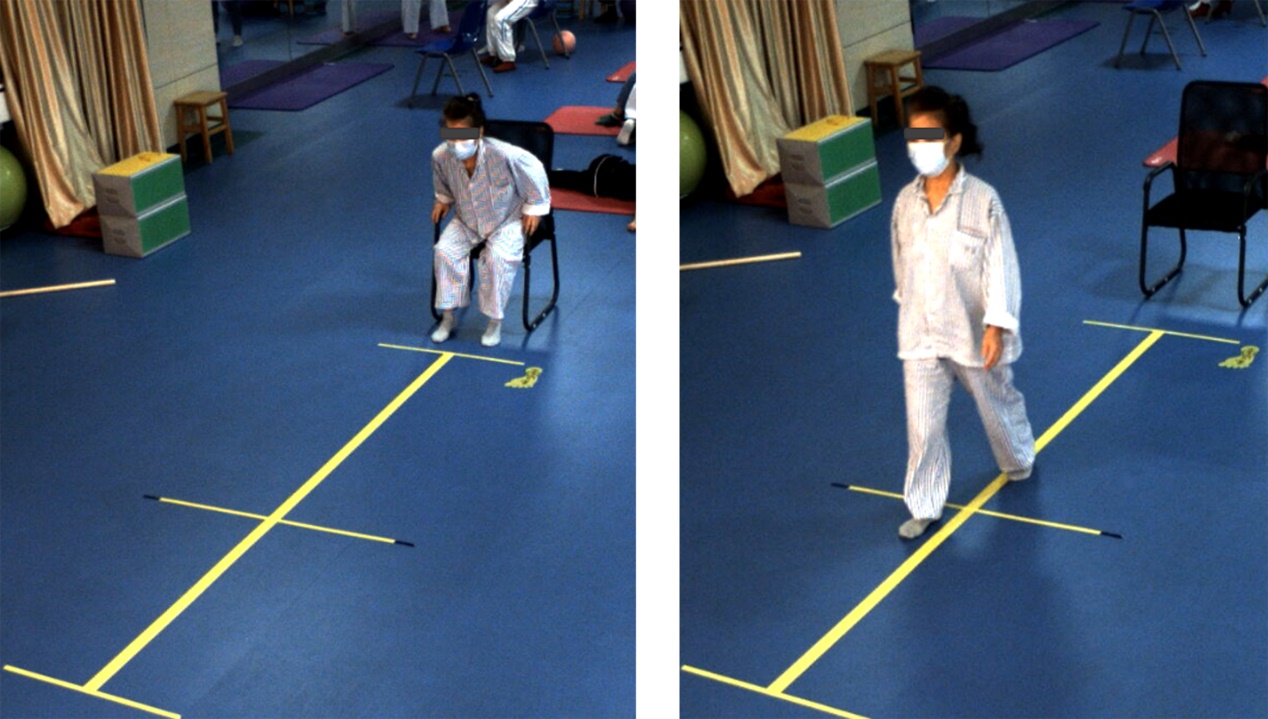


Test of daily activities (including sit-to-stand and walking)

# 2. Pictures of the data process through Fast-Move 3D Motion software


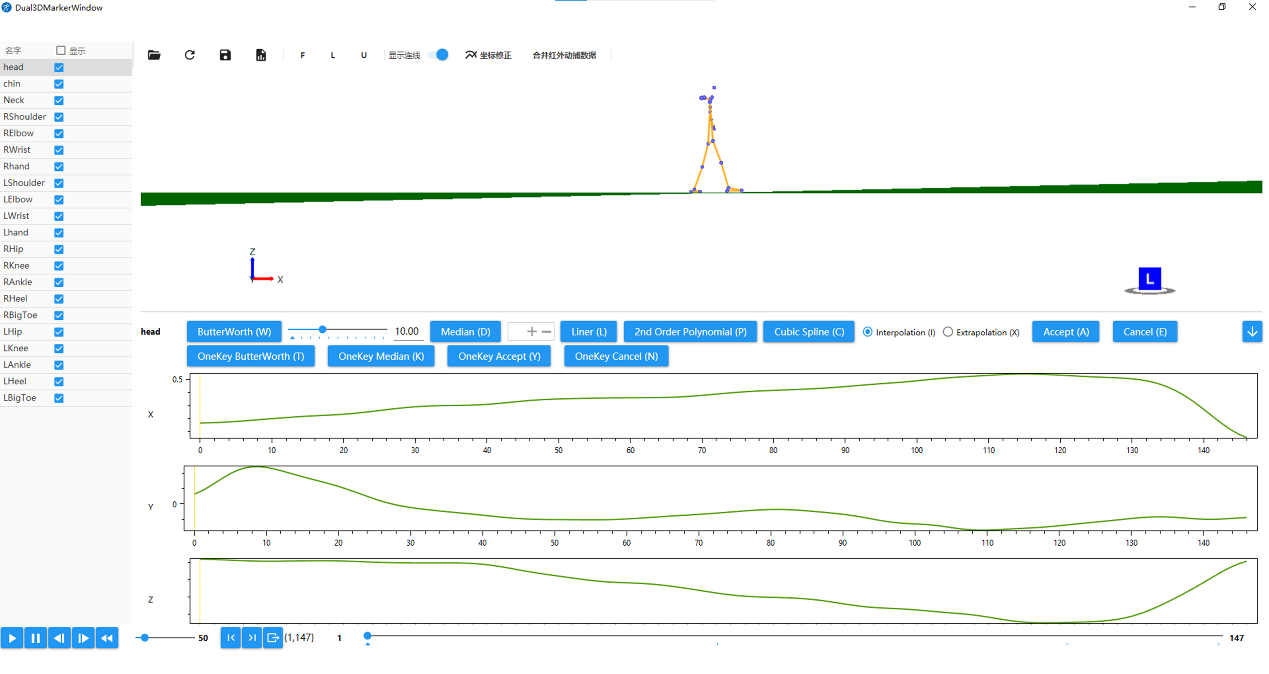


Three-dimensional coordinate trajectories of the 21 key body points

(The 21 key body points: head, chin, neck, bilateral shoulder, elbow, wrist, hand, hip, knee, ankle, heel and big toe.)


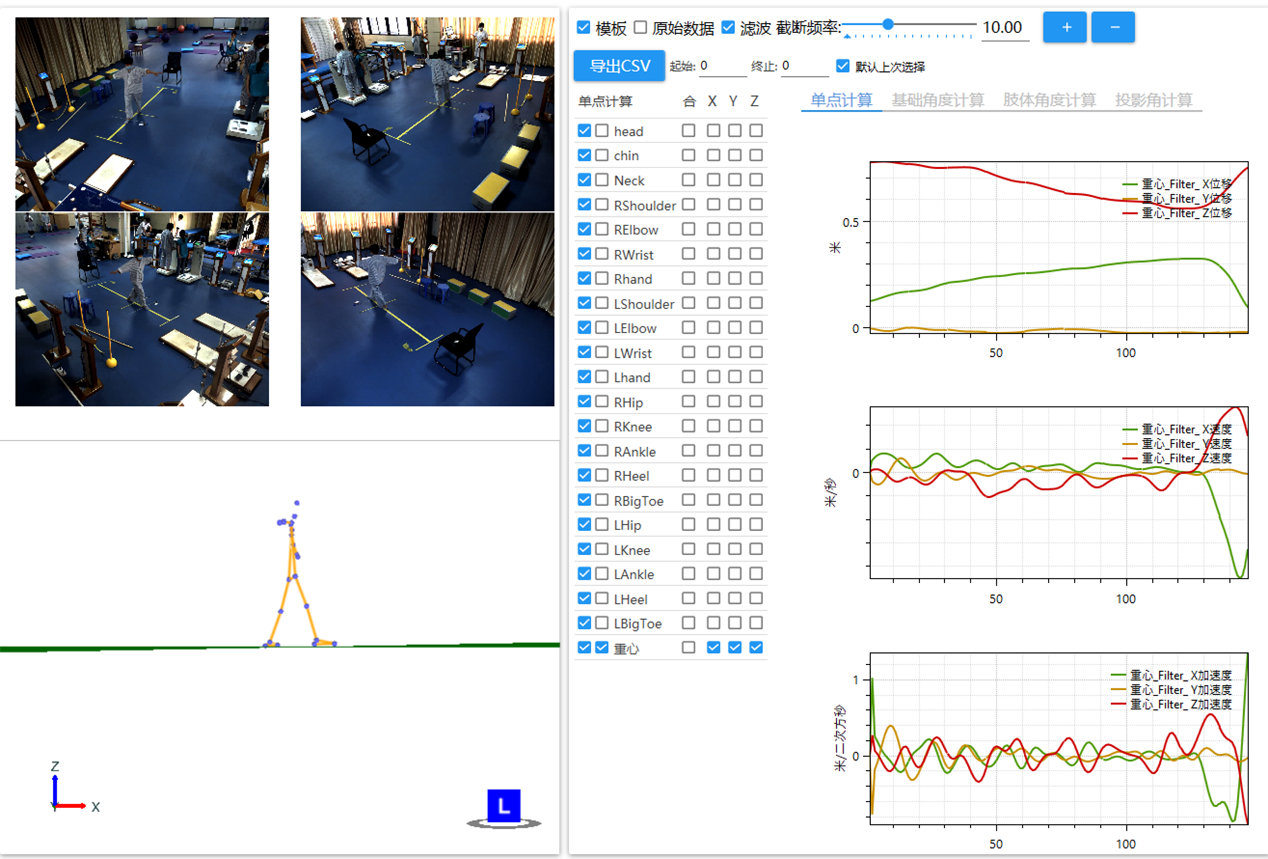


Calculation of the joint angles and three-dimensional coordinates of the centre of mass

# 3. Data processing

The trunk sagittal and frontal angles and COM ML displacement, which represent balance control, were taken as absolute values. Due to the high difficulty of the in-line lunge, some patients only partially completed this task, which affected the indices of balance control. Therefore, the in-line lunge data were further standardized by dividing by the peak knee flexion angle. The trunk sagittal angle in the sit-to-stand test was taken as the original value because it mainly reflects function rather than balance. Step length was normalized to the participant’s height.
